# Nerve Growth Factor Levels in Term Human Infants: Relationship to Prenatal Growth and Early Postnatal Feeding

**DOI:** 10.1155/2018/7562702

**Published:** 2018-12-23

**Authors:** David Sánchez-Infantes, Rubén Cereijo, Giorgia Sebastiani, Miriam Pérez-Cruz, Francesc Villarroya, Lourdes Ibáñez

**Affiliations:** ^1^Institut de Recerca Pediàtrica Hospital Sant Joan de Déu (IRP-HSJD), University of Barcelona, 08950 Esplugues, Barcelona, Spain; ^2^Institut d'Investigació en Ciències de la Salut Germans Trias i Pujol, 08916 Badalona, Barcelona, Spain; ^3^Department of Biochemistry and Molecular Biology, Institute of Biomedicine, University of Barcelona, 08028 Barcelona, Spain; ^4^Centro de Investigación Biomédica en Red Fisiopatología de la Obesidad y Nutrición (CIBEROBN), ISCIII, Madrid, Spain; ^5^Centro de Investigación Biomédica en Red de Diabetes y Enfermedades Metabólicas Asociadas (CIBERDEM), ISCIII, Madrid, Spain

## Abstract

**Background:**

Nerve growth factor (NGF) plays a key role in neuroprotection and developmental maturity. We assessed longitudinally the circulating concentrations of NGF in term healthy human newborns and infants as well as their association with prenatal growth and early postnatal feeding patterns.

**Methods:**

Circulating NGF and anthropometric measures (weight, length, body mass index, and ponderal index) were assessed longitudinally—at birth and at age 4 months—in 86 term infants born appropriate (AGA), small (SGA), or large for gestational age (LGA).

**Results:**

Cord blood NGF levels in SGA newborns were higher than those in AGA newborns (1.41 ± 0.2 pg/mL vs. 0.66 ± 0.1 pg/mL; *p* = 0.02) and not different from those in LGA neonates (0.79 ± 0.2 pg/mL). At age 4 months, SGA-breastfed infants showed the highest NGF concentrations (*p* = 0.02 and *p* = 0.01 vs. AGA and SGA-formula-fed infants, respectively), while LGA infants depicted a marginal increase. NGF levels in cord blood correlated negatively with the ponderal index at birth (*r* = −0.36; *p* = 0.0008).

**Conclusions:**

Circulating NGF is related to both prenatal growth and early postnatal nutrition. The maintenance of increased NGF concentrations in SGA-breastfed infants at age 4 months might be a potential mechanism to counterbalance potential risks for developing cognitive and psychomotor disadvantages.

## 1. Introduction

Nerve growth factor (NGF) is a protein belonging to the neurotrophin family [1]. Neurotrophins (NTs) play a role in neuroprotection [1–3], are of major importance in prenatal and postnatal brain development [4], and appear to play an important role in placental and fetal growth [5]. NGF and its precursor, pro-NGF, regulate neuronal development through cell survival and cell death signaling, acting through three types of receptors: TrkA, p75, and sortilin. The trophic effect of NGF is mediated through the TrkA and p75 receptors [6, 7], while pro-NGF induces apoptosis through the p75 receptor in conjunction with sortilin [8]. NGF is also important for the neuronal plasticity and survival of forebrain cholinergic neurons (cerebral cortex, hippocampus, basal forebrain, and hypothalamus), which are memory-related [9]. In addition, NGF plays a key role in memory and cognition; the decrease in NGF with aging could be involved in the age-dependent decline in those functions [10–13]. More recently, NGF has been reported to play a key role in angiogenesis [14].

Small-for-gestational-age (SGA) individuals have been reported to show poor academic performance, behavioral problems [15], and long-term cognitive impairments [16]. Multiple factors related to the postnatal environment, including childhood conditions, maternal mental health, and exposure to household cigarette smoking, among others, may be involved in this outcome [15]. Alterations in hippocampal formation and its related neural structures [17] as well as the type of early postnatal feeding (breast vs. formula) could also be implicated [18]. In the present study, we hypothesized that early modulation of NGF would relate to prenatal growth. To address this hypothesis, we prospectively assessed the developmental changes of NGF over the first 4 months of life in healthy term newborns born SGA, appropriate (AGA), or large for gestational age (LGA).

## 2. Materials and Methods

### 2.1. Study Population

**Figure 1:**
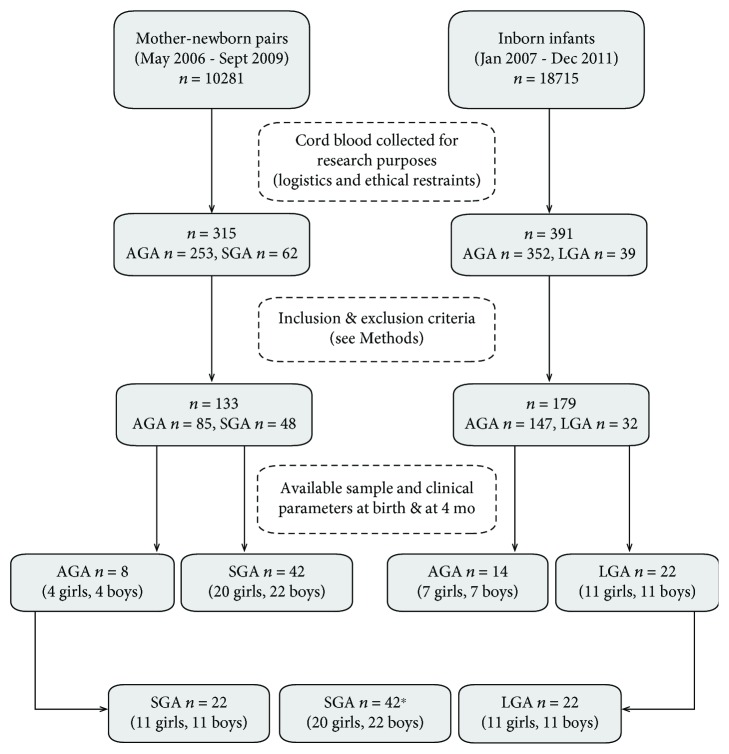
Recruitment of the study population. ^∗^Breastfed, *n* = 22 (11 girls, 11 boys); formula-fed, *n* = 20 (9 girls, 11 boys) (see text for details).

The study population consisted of *n* = 86 infants (42 SGA, 22 AGA, and 22 LGA) selected among those participating into two longitudinal studies that assess the body composition and endocrine-metabolic state of SGA and LGA infants, as compared to AGA controls, in the first postnatal years [19–22] (Figure 1). The present study focused on the comparison of NGF levels among AGA, SGA, and LGA infants at birth and at age 4 months. The inclusion and exclusion criteria have been described in previous studies; briefly, SGA, AGA, and LGA were, respectively, defined as having a birth weight *Z*-score below −2, between −1 and +1, and above +2 [19–22]. Approval by the Institutional Review Board of Barcelona University, Hospital Sant Joan de Déu, and written informed consent by the newborn's mother and/or father were obtained.

### 2.2. Anthropometry

Weight was measured with a beam balance (seca, Hamburg, Germany), and length was measured with a length board (seca 207; seca, Hamburg, Germany), using the mean of three measurements for analysis [21].

### 2.3. Sample Collection

Blood from the longitudinally studied infants was sampled at birth (from the umbilical cord before placental separation) and at age 4 months because this is the usual timing for switching to complementary feeding. At age 4 months, blood was sampled in the morning (between 8:00 and 9:00 am) and under fasting conditions. Serum was obtained after blood centrifugation for 10 minutes at 10000 rpm at 4°C. Serum aliquots were then stored at −80°C until further analysis.

### 2.4. NGF Assessment

Circulating NGF levels were determined using MILLIPLEX® MAP from Millipore (HADK2MAG-61K-01; Human Adipokine), as used in other previous studies [23–25]. This method is based on the Luminex xMAP technology, using proprietary techniques to internally color-code microspheres with two fluorescent dyes. Through precise concentrations of these dyes, 100 distinctly colored bead sets can be created, each of which is coated with a specific capture antibody. A maximum of 25 microlitres per well of serum was used for this assay. The detection limit for the NGF assay was 0.3 pg/mL. The intra- and interassay coefficients of variation (CVs) were 4% and 11%, respectively.

### 2.5. Statistics

Statistical analyses were performed using IBM SPSS Statistics 19.0 (IBM SPSS Inc., Chicago, IL). Results are expressed as mean ± SEM. Differences between groups were analyzed using the one-way ANOVA test followed by the Bonferroni test for comparisons between the 3 groups. Univariate associations of NGF with anthropometric parameters were tested by Spearman correlation followed by multiple regression analyses in a stepwise manner and corrected for confounding factors; *p* < 0.05 was considered statistically significant.

## 3. Results

Anthropometric parameters and serum NGF concentrations at birth and at 4 months are shown in Table 1.

### 3.1. Circulating NGF Levels in AGA, SGA, and LGA Newborns at Birth and at 4 Months

**Figure 2:**
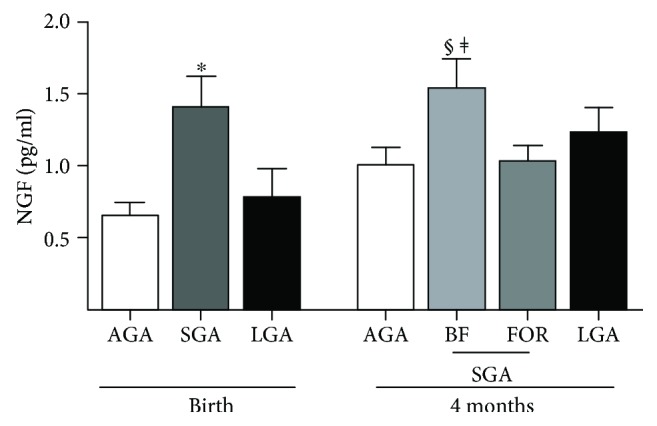
Developmental changes in circulating neural growth factor (NGF) in human infants born appropriate (AGA), small (SGA), or large for gestational age (LGA) both at birth and at age 4 months. AGA, *n* = 22; SGA, *n* = 42; LGA, *n* = 22. At 4 months, SGA infants are split into two groups according to the type of feeding from 0 to 4 months (SGA-breastfed (BF), *n* = 20; SGA-formula-fed (FOR), *n* = 22; see text for details). ^∗^*p* = 0.02 vs. AGA; ^§^*p* = 0.02 vs. AGA; ^‡^*p* = 0.01 vs. SGA-FOR.

At birth, serum concentrations of NGF in SGA newborns were significantly higher than those in AGA newborns and not different from those in LGA neonates (Figure 2). At age 4 months, serum NGF levels were similar in AGA and LGA infants and showed a significant increase compared to those in cord blood. In contrast, in SGA infants, NGF levels were similar to those at birth.

### 3.2. NGF Modulation at 4 Months in SGA Infants after Breastfeeding or Formula Feeding

When SGA infants were subdivided according to the type of feeding over the first 4 months of life (breastfed vs. formula-fed), SGA-breastfed infants had significantly higher levels of serum NGF than AGA infants and formula-fed SGA infants but comparable values to those of LGA infants. In SGA formula-fed infants, NGF levels at age 4 months were similar to those in AGA and LGA infants (Figure 1).

### 3.3. Correlations

NGF in cord blood correlated inversely with BMI (*r* = −0.38; *p* = 0.0004; *n* = 86) and ponderal index (PI) (*r* = −0.36; *p* = 0.0008; *n* = 86) in AGA, SGA, and LGA infants. These associations were independent from gestational age and gender.

## 4. Discussion

Here, we report for the first time circulating NGF levels in term AGA, SGA, and LGA human newborns from uncomplicated pregnancies, at birth and at age 4 months, and describe their association with early postnatal feeding patterns.

NGF has a major role in prenatal and postnatal brain development [26] and may also influence birth outcomes and neurodevelopmental risk through angiogenesis and cellular growth [27]. In addition, epigenetic mechanisms such as methylation or acetylation could also modulate NGF gene expression levels [28].

In our study, SGA infants born at term displayed—at birth—markedly higher NGF levels than AGA and LGA infants. In humans, the so-called critical brain growth spurt occurs between the third trimester of pregnancy and the second year postterm within appropriate environmental conditions [29]. This feature would explain the low cord blood NGF levels in preterm SGA newborns and in those delivered after pregnancies complicated with preeclampsia, gestational diabetes, or other disorders, where the timing of NGF secretion, release, and/or transport may be significantly affected [15, 28, 30]. In preterm deliveries, decreased NGF levels could also be explained by the presence of increased oxidative stress, which might disturb membrane fluidity and affect the NGF signaling cascade. Indeed, our data are in agreement with those of Kilari et al., who reported higher cord levels of NGF in normotensive mothers delivering SGA babies compared to those delivering normal-weight babies [27].

To our knowledge, no data on NGF levels in LGA newborns from nondiabetic mothers have been previously reported; our results show that in this population, NGF levels increase significantly from birth to 4 months. Moreover, at 4 months, NGF levels were comparable to those of AGA newborns, suggesting a normal early development of NGF in this population.

At age 4 months, SGA-breastfed infants maintained significantly higher circulating levels of NGF, while in formula-fed SGA infants, NGF concentrations decreased within the range of AGA and LGA infants. These differences could account, at least in part, for a poorer developmental quotient in formula-fed as compared to SGA-breastfed infants [18]. Previous studies have reported higher concentrations of trophic factors in human milk than in other biological fluids at different periods of maturation [31]. Thus, it is tempting to speculate that, given the role of NGF in neurodevelopment, the elevated NGF levels in SGA-breastfed infants could reflect the presence of a higher amount of NGF in breast milk, acting as a compensatory mechanism aimed at preserving and/or improving the cognitive function in those children. Indeed, SGA-breastfed infants have been reported to show higher Bayley Mental Development Index and Psychomotor Development Index as compared to formula-fed SGA infants [18]. This compensatory mechanism would not be operational in those cases where prenatal growth restraint associates to maternal preeclampsia [32].

At birth, NGF showed an inverse correlation with body weight and ponderal index in all subjects. Other investigators have also reported this association in newborns from normotensive healthy mothers [27]. A negative correlation between brain-derived neurotrophic factor cord blood levels and birth weight was also described in a population of AGA, SGA, and LGA babies [33].

Overall, these results may provide novel insights on the mechanisms accounting for the neurodevelopment adaptation of SGA infants and on the potential higher risk of these individuals to develop cognitive and psychomotor disadvantages. The main limitations are the lack of follow-up beyond age 4 months; the absence of measurements of other neurotrophic factors, for example, brain-derived neurotrophic factor, NT-3, and NT-4; and the lack of specific cognitive and psychomotor prospective assessments in the study subjects. In addition, we were not able to measure placental NGF concentrations, to ascertain their contribution to cord NGF or to assess maternal NGF levels, which nevertheless, have been found to be decreased in preterm vs. term pregnancies and in preeclamptic vs. normotensive women [28, 34]. Moreover, since NGF is considered to be an endocrine factor and its circulating levels are modulated under pathological conditions [27, 28, 34], the analysis of mRNA expression of this factor at tissue level does not appear to be so relevant in this context. Further studies evaluating the relationships between breast milk NGF concentrations, duration of lactation, and long-term cognitive and psychomotor outcome of SGA subjects are needed to decipher the precise role of neurotrophic factors in neurodevelopment.

## 5. Conclusions

In summary, we report for the first time NGF concentrations in healthy term newborns with a broad range of birth weights from uncomplicated pregnancies at birth and at age 4 months. We disclose that NGF levels are higher in SGA newborns at birth and are maintained within the same range at 4 months only in those receiving exclusive breastfeeding.

## Figures and Tables

**Table 1:** Anthropometric variables and serum nerve growth factor levels at birth and at 4 mo in the study population.

	AGA			SGA			LGA		
	At birth	At 4 mo	Δ0–4 mo	At birth	At 4 mo	Δ0–4 mo	At birth	At 4 mo	Δ0–4 mo
*n*	22	22	22	42	42	42	22	22	22
Girls (*n*, %)	11 (50)	11 (50)	11 (50)	20 (47.6)	22 (52)	21 (50)	11 (50)	11 (50)	11 (50)
Cesarean section (*n*, %)	5 (22)	—	—	7 (16)	—	—	9 (41)	—	—
Gestational age (w)	39.9 ± 0.2	—	—	38.8 ± 0.2^b^	—	—	40.2 ± 0.3^¶^	—	—
Weight (kg)	3.3 ± 0.1	6.8 ± 0.1	3.5 ± 0.2	2.3 ± 0.03^c^	5.9 ± 0.1^b^	3.6 ± 0.1	4.2 ± 0.1^c,¶^	7.7 ± 0.1^b,#^	3.15 ± 0.1
Weight *Z*-score	−0.04 ± 0.1	−0.2 ± 0.2	−0.1 ± 0.2	−2.3 ± 0.1^c^	−0.76 ± 0.1^a^	1.7 ± 0.1^a^	2.5 ± 0.1^c,¶^	1.3 ± 0.3^b,¶^	−1.2 ± 0.3^a,‡^
BMI (kg/m^2^)	14.2 ± 0.3	17.2 ± 0.3	2.9 ± 0.4	11.6 ± 0.2^b^	16.3 ± 0.2^a^	4.86 ± 0.3^b^	15.3 ± 0.3^b,#^	18.3 ± 0.5^¶^	3.0 ± 0.7^‡^
PI (kg/m^3^)	27.6 ± 0.5	27.7 ± 0.9	−0.3 ± 0.8	24.8 ± 0.5^b^	26.5 ± 0.4	2.0 ± 0.9^a^	28.3 ± 0.7^#^	28.3 ± 1.1	−0.03 ± 1.3
NGF (pg/mL)	0.66 ± 0.3	0.99 ± 0.1^∗^	0.33 ± 0.2	1.41 ± 0.2^a^	1.60 ± 0.2	−0.19 ± 0.2	0.79 ± 0.2	1.24 ± 0.2^∗∗^	0.41 ± 0.2

Values are mean ± SEM. BMI: body mass index; PI: ponderal index; NGF: nerve growth factor. ^a^*p* = 0.02, ^b^*p* ≤ 0.001, and ^c^*p* ≤ 0.0001 vs. AGA, ^‡^*p* < 0.05, ^#^*p* ≤ 0.001, ^¶^*p* ≤ 0.0001 vs. SGA, ^∗^*p* = 0.02 vs. at birth; ^∗∗^*p* = 0.01 vs. at birth.

## Data Availability

That data will not be shared, because the data presented in this article are partial information from a larger study.
